# Canal to diaphysis ratio as a risk factor for hip fractures and hip fracture pattern

**DOI:** 10.1051/sicotj/2017051

**Published:** 2017-11-10

**Authors:** Prasad Ellanti, Kunal Mohan, Andrew Moriarity, Niall Hogan, Tom McCarthy

**Affiliations:** 1 Department of Trauma & Orthopaedics, Saint James’s Hospital James’s Street Dublin DO8NHY1 Ireland

**Keywords:** Canal-diaphysis ratio, Osteoporosis, Hip fractures

## Abstract

*Introduction*: Osteoporosis and related fractures constitute a significant burden on modern healthcare. The standard method of diagnosing osteoporosis with a dual-energy X-ray absorptiometry (DXA) scan is limited by accessibility and expense. The thickness of the cortex of the proximal femur on plain radiographs has been suggested to be a method for indicating osteoporosis and as a risk factor of hip fractures in the elderly.

*Methods*: A retrospective study was undertaken to assess the usefulness of the canal-diaphysis ratio (CDR) as a risk factor for developing a hip fracture, excluding patients presenting under 50 years old, following high-energy trauma or pathological fractures. The CDR was measured in 84 neck of femur (NOF) fracture patients and 84 intertrochanteric hip fracture patients, and these were subsequently compared to the CDR of 84 patients without a hip fracture. Measurements were taken on two occasions by two members of the orthopaedic team, so as to assess the test’s inter- and intraobserver reliability.

*Results*: In comparison to those without a fracture, there was a significant difference in the CDR of patients with a NOF fracture (*P* < 0.0001) and intertrochanteric fracture (*P* < 0.0001). Furthermore, the odds of having a CDR above 60.67 and 64.41 were significantly higher in the NOF (OR = 2.214, *P* = 0.0129) and intertrochanteric fracture (OR = 32.27, *P* < 0.0001) groups respectively, when compared to the non-fractured group. The analysis of the test’s inter- and intraobserver reliability showed strong levels of reproducibility.

*Discussion*: We concluded that a raised CDR was associated with an increased incidence of NOF and intertrochanteric hip fracture. Measuring the CDR can thus be considered as a reproducible and inexpensive method of identifying elderly patients at risk of hip fractures.

## Introduction

Osteoporosis is the most common disease affecting bone in humans [1] with over 200 million people affected worldwide [2]. It is characterised by reduced bone mass and deterioration of the bone structure, subsequently increasing the risk of associated fracture. The World Health Organization has defined osteoporosis as a bone mineral density (BMD) value more than 2.5 standard deviations (*SD*) below the mean for the young adult reference population. In the context of increasing life expectancy globally, the incidence of osteoporotic hip fracture is expected to rise from 1.66 million in 1990 to 6.26 million by 2050 [3]. The National Institute for Health and Clinical Excellence (NICE) reports that direct medical costs in the UK as a result of fragility fractures have been estimated at 2.3 billion pounds, and are expected to rise to six billion pounds by 2030, with the majority of costs relating to hip fracture care [4]. Furthermore, hip fractures themselves have been shown to be a source of significant morbidity and mortality amongst the elderly population [5, 6]. Despite the well-documented burden and morbid sequelae of osteoporosis, up to 75% of those suffering from the condition remain undiagnosed [7]. Given this, an increased focus has been placed on the diagnosis and prevention of the disease and associated fractures, allowing prompt initiation of any required prevention measures or pharmacological therapy, thereby maximising the quality of life in this growing elderly patient cohort [8].

Presently, the current gold standard, and the most widely adopted method for diagnosis of osteoporosis, is the dual-energy X-ray absorptiometry (DXA) scanning [9–11]. There are however a number of limitations on the utility of DXA scanning in everyday practice. The use of DXA scanning can be limited by the cost, availability and portability, particularly in the context of units with limited resources. Furthermore, osteoporotic fractures are multifactorial [12], with reduced BMD being just one factor that contributes to an increased risk of fracture. Other factors, such as the structural and elastic properties of the bone are not taken into account by modalities such as the DXA [13]. As a result, alternative methods of diagnosing osteoporosis and fracture risk need to be explored.

The thickness of the cortex of the proximal femur on plain radiographs has been suggested as an alternative method for indicating osteoporosis and as a risk factor of hip fractures in the elderly. It has been suggested that femoral cortex thickness can be quantified through obtaining the ratio of the femoral canal and diaphysis width, the hypothesis being that the higher the ratio, the wider the canal, and hence thinner the femoral cortex, making the bone itself more structurally susceptible to fracture. The primary objective of our study was thus to evaluate the usefulness of this canal-diaphysis ratio (CDR) as a risk factor for developing osteoporotic hip fractures. Additionally, the secondary objective was to assess whether there is any difference in the utility of measuring the CDR across different hip fracture types.

## Materials and methods

A retrospective, observational case-control study of plain radiographs of patients presenting to the authors’ institution between February 2007 and August 2015 was undertaken. The radiographs were divided into two age and gender-matched groups, namely a study group (presenting with radiological evidence of the hip fracture) and a control group (with no radiographic evidence of the current or past hip fracture).

The study group was subdivided into the following cohorts:

1. Eighty-four patients presenting with a neck of femur (NOF) fracture (AO classification 31B1–31B3) on plain radiograph, with a mean age of 79.4 years (*SD ±* 9.21). This group consisted of 42 males and 42 females, with a mean age of 77.3 (*SD ±* 8.915) and 81.5 (*SD ±* 9.14) years, respectively.
2. Eighty-four patients presenting with an intertrochanteric hip fracture (AO classification 31A1–31A3) on plain radiograph, with a mean age of 78.1 years (*SD ±* 10.8). This group consisted of 42 males and 42 females, with a mean age of 74.6 (*SD ±* 9.4) and 81.54 (*SD ±* 11.1) years, respectively.

Each study group was compared to a control group of 84 patients, with a mean age of 74.6 years (*SD* ± 10.8). This group consisted of 42 males and 42 females, with a mean age of 72.7 (*SD* ± 10.1) and 76.6 (*SD* ± 11.2) years, respectively. All patients presenting under the age of 50 years old or following high-energy trauma or pathological fractures were excluded from the study.

All radiographs were performed on presentation to the authors’ institution, using a standardised imaging approach. Each patient was identified through their Medical Record Number (MRN), and all patient radiographs and demographics were subsequently obtained using the institution’s Electronic Patient Record (EPR) system. Patients presenting with AP hip radiographs that showed suboptimal penetrance, or in which the lesser trochanter of the femur was difficult to delineate, were also excluded from this study.

Each radiograph was measured electronically using annotation tools on the Cerner ProVision^®^ System (Markham, Canada). In the patients presenting with a first NOF or intertrochanteric fracture, the contralateral, intact hip was used for measurement, so as to avoid errors resulting from any potential external rotation of the fractured limb. In the control group, one of the hips was chosen for measurement. The technique used to measure the canal-diaphysis ratio of each of the radiographs in both the study and control groups which is based on the canal-to-calcar ratio was described by Dorr et al. [14], where a horizontal line at the mid-lesser trochanter was drawn on the anteroposterior (AP) hip radiograph. Sah et al. [15] measured cortex thickness at a point 10 cm distal and parallel to the mid-lesser trochanteric line on both AP and lateral views and found a correlation between DXA *t*-scores and cortical thickness. They suggested a cortical thickness index of ≤ 0.4 should warrant referral for osteoporosis evaluation and treatment.

**Figure 1.**
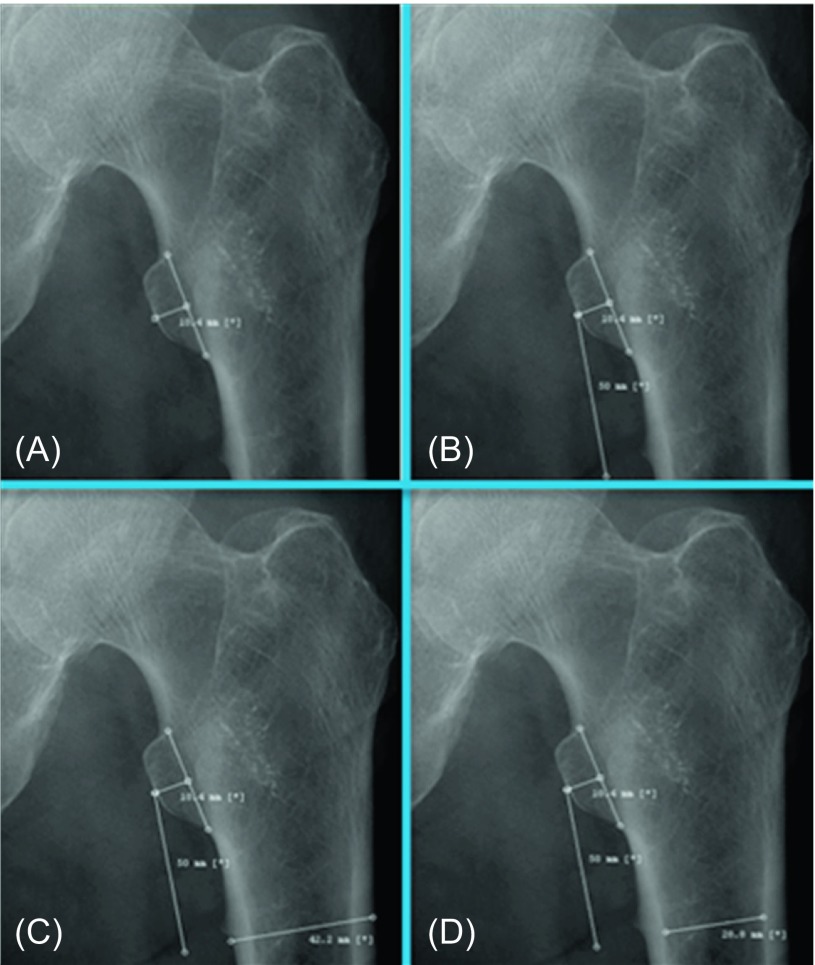
The method for measuring the canal-diaphysis ratio (CDR).

Our method of measuring the CDR was similar to those of both Sah and Dorr et al., and is illustrated in Figure 1. First, we identified the midpoint of the lesser trochanter on the AP view of the hip (Figure 1A). From here, a 5 cm line was formed distal to this point, running parallel with the femoral shaft (Figure 1B). At this point, perpendicular lines were created that intersected the femur transversely, to establish the width of the femoral diaphysis (Figure 1C) and canal (Figure 1D). The CDR for each radiograph was subsequently determined using the obtained ratio of these two values.

Measurements were taken by a member of the orthopaedic team, and subsequently taken again by the same member as well as another member, in order to evaluate the inter- and intraobserver reliability of the screening tool.

Statistical analysis was performed using SPSS version 11.5 for Windows^®^. The normality of the data distribution was initially tested using both Kolmogorov-Smirnov and Shapiro-Wilk analyses, which showed a normal distribution. The mean values of each group were subsequently compared by independent *T*-test and when *P* values were less than 0.05, the results were considered statistically significant [16]. Furthermore, odds ratios (OR) were calculated at the mean CDR of each fracture group in comparison to the non-fractured patients, so as to further delineate any potential relationship between the fracture and raised CDR. The results were considered significant with an OR > 1 and *P* value less than 0.05 [16].

The intra- and interobserver reproducibility was evaluated by comparing the mean canal-diaphysis ratios, and the intraobserver reliability was assessed using Pearson’s correlation coefficient, with a value of above 0.6 considered to signify a strong correlation [17]. The interobserver reliability was calculated by determining the intraclass correlation coefficient (ICC) value, using a two-way mixed average measure model (absolute agreement) and the 95% confidence interval (95% CI). The ICC was considered to be in excellent agreement when the ICC value was ≥ 0.75 and between fair and good when the ICC value was between 0.4 and 0.75 [16].

## Results

The patient demographics of each of the hip fracture groups and control patients are listed in Table 1. The mean CDR values are shown in Table 2. Overall, the mean CDR value is slightly less in males compared to females when comparing the fractured and non-fractured groups as a whole, as well as within the NOF and intertrochanteric hip fracture groups themselves. Of the hip fracture patients the lowest mean CDR (59.71 (*SD ±* 7.620)) is seen in the male NOF fracture group, while the highest mean CDR (94.41 (*SD ±* 7.489)) is seen in the female intertrochanteric hip fracture group. Statistical analysis of the mean CDR values (Table 2) shows a statistically significant (*P* < 0.0001) difference in the mean CDR for the NOF and intertrochanteric fracture cohorts in comparison to the non-fractured control patients. Furthermore, the subdivision of the NOF and intertrochanteric fracture cohorts by sex also shows a statistically significant difference (*P* < 0.05) in the CDR when compared to their non-fractured sex-matched counterparts.

**Table 1.** Demographics.

Fracture type	Neck of femur	Intertrochanteric	No fracture
Number (F:M)	84 (42:42)	84 (42:42)	84 (42:42)
Mean age in years (F:M)	79.4 (81.48:77.34)	78.1 (81.54:74.62)	74.6 (76.64:72.48)

**Table 2.** Mean canal-to-diaphysis ratios.

Fracture type	Mean CDR fracture	Mean CDR no fracture	*P* value
Neck of femur	60.670 (*SD* ± 7.585)	55.70 (*SD* ± 7.527)	< 0.0001
Male	59.710 (*SD* ± 7.620)	55.08 (*SD* ± 7.279)	0.0055
Female	61.628 (*SD* ± 7.518)	56.32 (*SD* ± 7.806)	0.0021
Intertrochanteric	64.410 (*SD* ± 7.489)	55.70 (*SD* ± 7.527)	< 0.0001
Male	63.690 (*SD* ± 7.826)	55.08 (*SD* ± 7.279)	< 0.0001
Female	65.130 (*SD* ± 7.155)	56.32 (*SD* ± 7.806)	< 0.0001

Table 3 illustrates the OR calculated for the mean CDR of each fracture pattern group in comparison to the non-fractured cohort. The odds of having a CDR above 60.67 and 64.41 were significantly higher in the NOF (OR = 2.214, *P =* 0.0129) and intertrochanteric (OR = 32.27, *P* < 0.0001) hip fracture groups, respectively, when compared to the non-fractured group. When comparing the NOF fracture and non-fractured cohorts, 60% of the patients measured to have a CDR of ≥ 60.67 had a fracture, in comparison to only 44% in patients with a CDR of < 60.67. Similarly, when comparing the intertrochanteric and non-fractured cohorts, 95% of the patients measured to have a CDR ≥ 64.41 were presenting with an intertrochanteric fracture, compared to only 36% in patients with a CDR < 64.41.

**Table 3.** Odds ratios (versus no fracture group).

Fracture type	Mean CDR fracture	OR	*P* value
Neck of femur	≥ 60.67	2.214	0.0129
Intertrochanteric	≥ 64.41	32.270	< 0.0001

Table 4 illustrates the inter- and intraobserver scores with intraclass correlation (ICC) and Pearson’s correlation coefficients, respectively. The ICC shows a good agreement in measurements between measurers in both fractured and non-fractured groups, and the Pearson’s correlation coefficients correspond to a strong reproducibility of measurements within each measurer in both the fractured and non-fractured cohorts.

**Table 4.** Inter- and intraobserver reliability.

Group	ICC	Pearson’s correlation coefficient	
		Observer 1	Observer 2
Hip fracture	0.725 (0.70–0.74)	0.7496	0.6922
No fracture	0.696 (0.67–0.72)	0.7344	0.6811

## Discussion

The burden of osteoporosis is increasing with over 200 million people worldwide with the disease. A large number of these patients remain undiagnosed. The gold standard for the diagnosis of osteoporosis and the evaluation of fracture risk is not universally accessible due to factors such as cost, availability, portability and the inherent dangers of ionising radiation. Furthermore, in the context of hip fractures in an ageing population, BMD is one of the many factors, such as bone geometry, elasticity and bone turnover which contribute to fracture risk [18]. Changes in the proximal femur geometry with age, in particular with women, have shown to increase the mechanical stresses at the femoral neck increasing the likelihood of fractures [19]. These findings all provide the supporting evidence for the potential limitations and practical utility of DXA scanning in detecting patients at risk of osteoporotic hip fractures.

The plain radiograph of the hip, on the other hand, is a simple and frequently performed investigation. It has the potential to diagnose multiple pathologies including degenerative joint disease and fractures. Measuring osteoporosis on plain film radiographs of the hip has previously been proposed by the Singh index [20], with some subsequent studies having shown the correlation between the Singh index and osteoporosis, others not having found any correlation [21]. The study by Sah et al. [15] found a correlation between the proximal femur Dorr type, the cortical thickness index and *T*-scores. The existence of these studies in the literature may help to provide further evidence for the potential utility of using a simple plain radiograph in the context of detecting patients at risk of osteoporosis-related hip fractures, and this, coupled with the findings above and anecdotal evidence of differences in intraoperative femoral bone quality, formed the basis of the rationale for performing our study.

While studies similar to ours have been done in the past [15, 20], we believe that our study is the first to comprehensively evaluate the utility of measuring the femoral cortical thickness across different common hip fracture patterns. Differences in CDR in our study were significant across both studied fracture groups when compared to a non-fractured normal cohort. This supports the theory that measuring a simple ratio of the proximal femoral diameter can provide a useful screening tool for identifying patients at risk of hip fracture. These cohorts, well matched both for sex and age further strengthen our hypothesis. Furthermore, the levels of inter- and intraobserver reproducibility detected in our study support the potential validity and reproducibility of this measuring tool should it be applied in a clinical setting.

Our study is a non-randomised retrospective review, and as such has inherent limitations. The small numbers involved also further limit the clinical significance and application of its findings, and while attempts were made to match each studied patient cohort as closely as possible in regard to both sex and age, group heterogeneity in other domains can also impact ultimate significance. Lastly, despite all scans being performed in a single centre under the same protocol with good levels of reproducibility being detected in our findings, assumed bias associated with both imaging standards and measurements could theoretically also impact upon the true validity of our results in this context.

Moving forward, studies would ideally be prospective in nature and with larger numbers, and in particular would require correlation of the mean CDR values to corresponding DXA scores to further provide evidence for the validity of this tool. This approach may help establish a validated CDR measuring tool and potentially allow the establishment of a true “cut-off” CDR which would identify a patient at high risk of osteoporosis-related hip fractures. Identification of this at an early stage would help instigate appropriate management and potentially reduce the chance of a fragility-related hip fracture. The 2007 Blue Book published jointly by the British Orthopaedic Association and British Geriatrics Society gives guidelines on the care of patients with fragility. This includes the need for patients presenting with a fragility fracture to be assessed for the requirement of antiresorptive therapy [22]. A further validated CDR measurement tool may aid in effective and prompt implementation of this clinical guideline.

In conclusion, we found that measuring the CDR on plain film radiographs is quick, easy and reproducible. We have shown that a raised CDR is associated with an increased incidence of NOF and intertrochanteric hip fracture. Measuring the CDR can thus be considered as a reproducible and inexpensive alternative to DXA scanning in identifying elderly patients at risk of hip fractures.

## Conflict of interest

The authors declare that they have no conflict of interest in relation with this paper.
